# Public Transportation and Pulmonary Tuberculosis, Lima, Peru

**DOI:** 10.3201/eid1310.060793

**Published:** 2007-10

**Authors:** Olivia J. Horna-Campos, Héctor J. Sánchez-Pérez, Inma Sánchez, Alfredo Bedoya, Miguel Martín

**Affiliations:** * Universidad Autónoma de Barcelona, Barcelona, Spain; † Colegia de la Frontera Sur, Chiapas, Mexico; ‡ Dirección de Salud IV Lima Este, Lima, Peru

**Keywords:** Tuberculosis, pulmonary tuberculosis, public transportation, minibuses, poverty, Peru, dispatch

## Abstract

The association between public transportation for commuting and pulmonary tuberculosis (TB) was analyzed in workers in Lima, Peru. Traveling in minibuses was a risk factor for pulmonary TB. Preventive measures need to be taken by health services to prevent spread of this disease.

Tuberculosis (TB) continues to be an important public health problem in impoverished areas (*1**–**4*). It is spread through the air by patients with pulmonary TB (*5*). Because those most affected by pulmonary TB are persons 15–50 years of age, employment-related characteristics of these persons must be taken into account when studying this disease. In Lima, Peru, residents of peripheral neighborhoods generally use minibuses to travel to work or school and have long commute times. Because public transportation in Latin America routinely carries more passengers than permitted by law, it is plausible to assume that in areas with endemic pulmonary TB, daily use of public transportation may be a risk factor for acquiring TB (*6**–**9*).

The greatest amount of expectoration (productive coughing) occurs during the morning commute (6:00 am–7:00 am) because of accumulation of bronchial secretions at night (*10*). Given the conditions in which persons travel to work in Lima (long travel times and overcrowding on minibuses with closed windows), we analyzed whether use of minibuses was associated with the spread of pulmonary TB as part of a larger study to assess pulmonary TB in the Ate-Vitarte District of this city.

## The Study

The study was conducted in the Ate-Vitarte district (population 365,473), which is located ≈12 km from the center of Lima. It is a marginal urban area that receives immigrants who come to Lima with high rates of TB. The study was reviewed and approved by the ethics committee of the East Lima Health Directorate IV.

During July–August 2004, interviews were conducted with a random sample of 150 commuters >15 years of age who had productive coughs for >15 days and who came to health services for treatment. A total of 142 persons agreed to participate: 96 were treated at hospitals and 46 were treated at health centers. Informed consent was obtained from all participants. Interviews were conducted in the health services that persons visited. We obtained demographic and socioeconomic information, as well as information on perceived health, and means of transportation used in commuting.

All persons with productive coughs were requested to provide 3 sputum samples (the first immediately after the interview and the other 2 on 2 consecutive days) for smear testing. Samples were tested by using the Ziehl-Neelsen method, which is used in all epidemiologic surveillance in Peru (*11*). Study participants were considered positive for pulmonary TB if >1 acid-fast bacilli (AFB) were found (*11*).

Results were analyzed by using bivariate and multivariate logistic models with SPSS version 12 software (SPSS Inc., Chicago, IL, USA). Statistical significance was defined as p<0.05.

Demographic and socioeconomic characteristics of the study group are shown in Table 1. Variables analyzed for persons tested for pulmonary TB are shown in Table 2. Seventeen (11.9%) of 142 study participants were smear positive (29.6% 1 cross, 41.0% 2 crosses, and 29.4% 3 crosses). Two persons had been discharged from a TB treatment program <6 months earlier.

**Table 1:** Demographic and socioeconomic characteristics of 142 persons tested for tuberculosis, Lima, Peru

Characteristic	Value
Women, %	55.6
Mean age, y	35.7
Indigenous (Quechua), %	38.7
15–44 years of age, %	77.5
Immigrants, %	60.6
Education, %	
Illiterate	8.5
Primary school	20.4
Secondary school or higher	71.1
Household indicators, %	
Only 1 room	18.3
Roof of solid material	41.5
Electricity	97.8
Connection to public water supply	56.3
Toilet with running water	58.5
Overcrowded conditions*	39.4
Social security	15.5
Occupation, men (n = 63), %	
Street peddlers	68.3
Students	17.5
Another job	12.7
Not working	1.6
Occupation, women (n = 79), %	
Street peddlers	39.2
Housewives	43.0
Students	13.9
Another job	3.8
Means of transportation, %	
Public transportation	45.7
Individual transportation	26.8
Do not travel to work	27.5
Time spent commuting,† %	
30 min to 1 h	60.0
>1 h	40.0

*More than 3 persons sleeping in the same room. †Of those using public transportation, n = 65.

**Table 2:** Variables analyzed in 142 persons tested for pulmonary tuberculosis, Lima, Peru*

Variable	AFB smear–positive, n/N (%)	AFB smear–negative, n/N (%)	Total, n/N (%)	OR (95% CI)	Positive prevalence ratio
Occupation away from home	16/17 (94.1)	87/125 (69.6)	103/142 (72.5)	6.99 (0.89–54.61)	6.06
Commuter transport by minibus	14/16 (87.5)	51/87 (58.6)	65/103 (63.1)	4.9 (1.06–23.09)	4.09
Commuting time > 1 h	8/16 (50)	20/87 (23)	28/103 (27.2)	3.35 (1.12–10.10)	2.07
History of pulmonary tuberculosis	4/17 (23.5)	24/125 (19.2)	28/142 (19.7)	1.29 (0.38–4.33)	1.25
Previous contact with tuberculosis cases (family)	7/17 (41.2)	40/125 (32.0)	47/142 (33.1)	1.49 (0.53–4.20)	1.41
Overcrowded conditions	6/17 (35.3)	50/125 (40)	56/142 (39.4)	0.818 (0.28–2.35)	0.83

*AFB, acid-fast bacilli; OR, odds ratio, CI, confidence interval.

None of the demographic and socioeconomic indicators analyzed (Table 1) were associated with pulmonary TB. Table 2 shows crude associations between variables and pulmonary TB. The use of a minibus and a commuting time >1 hour had the highest associations with pulmonary TB. Adjusted relationships obtained by logistic regression that controlled for all variables shown in Table 2 confirmed that commuting to work by minibus was associated with a positive test result for pulmonary TB (adjusted odds ratio 4.90, 95% confidence interval 1.04–23.04).

## Conclusions

We observed a pulmonary TB prevalence rate of 12% in persons with chronic productive coughs who came to health services in the study area. This rate was similar to a prevalence of 11% reported in a similar study conducted in an area of poor socioeconomic status in Chiapas, Mexico (*12*).

The proportion of persons 15–44 years of age with pulmonary TB in our study was consistent with data of the World Health Organization and the Peruvian Ministry of Health, which show that this age group has the highest prevalence of this disease (*5**,**13**,**14*). Our results also showed that there were no sex-related differences in the frequency of pulmonary TB (*13**, **14*).

Socioeconomic variables showed no association with pulmonary TB. However, this finding should be interpreted cautiously because of the small sample size, particularly the number of persons who lived in extreme poverty. Another factor that could limit our conclusions is accessibility of persons in areas of extreme poverty to public transportation. The fact that the field work phase of our study could not be increased because of shortages of resources and health center personnel time is also a limitation.

The relationship of having pulmonary TB with working at home or away from home showed a positive prevalence ratio of 6.06. Among persons working outside the home, commuting by minibus increased the risk of having pulmonary TB by a factor of 4.09 compared with persons who used individual forms of transportation. A commuting time >1 hour on a minibus also increased the risk for pulmonary TB by a factor of 2.07 (Table 2).

Minibuses in Lima increase the risk for pulmonary TB because they are usually overloaded (capacity is often doubled) in the early morning and late evening. Overcrowding, exposure to persons with productive coughs while commuting 2 times a day 5 days a week, and closed windows on minibuses, combined with a high prevalence of pulmonary TB in Lima, increase the risk of acquiring this disease. Because persons with cases of pulmonary TB have more productive coughs in the morning (when more bacilli are released because of their accumulation at night), there is increased risk for transmission of TB to other passengers (*15*), as has already been suggested by other studies in developing and industrialized countries (*6**–**9*). The findings that 41% of persons tested were positive with 2 crosses and 29.4% were positive with 3 crosses indicate poor TB prevention and control programs in the study area and higher probabilities of transmission (*15*).

Despite the limitations of our study, commuting in minibuses was a risk factor for pulmonary TB. Consequently, preventive measures need to be taken by health services to encourage persons with productive coughs to avoid this type of public transportation and to come to health services for diagnosis and treatment. Health services should also be more accessible to persons with pulmonary TB who, for whatever reason, cannot use other forms of transportation. This increased accessibility would include home treatment during the infectious phase of this disease.
